# Whole-Body Vibration for Individuals with Reconstructed Anterior Cruciate Ligament: A Systematic Review

**DOI:** 10.1155/2020/7362069

**Published:** 2020-05-01

**Authors:** Adérito Seixas, Borja Sañudo, Danúbia Sá-Caputo, Redha Taiar, Mário Bernardo-Filho

**Affiliations:** ^1^Escola Superior de Saúde, Universidade Fernando Pessoa, Porto, Portugal; ^2^Universidad de Sevilla, Sevilla, Spain; ^3^Universidade do Estado do Rio de Janeiro, Rio de Janeiro, Brazil; ^4^Université de Reims, France

## Abstract

**Background:**

ACL ruptures are a prevalent condition, affecting daily living activities, associated with high financial burden.

**Objective:**

To assess the effect of whole-body vibration (WBV) in the rehabilitation of patients with reconstructed anterior cruciate ligament. *Methodology*. An electronic search in Pubmed, Scopus, Web of Science, and PEDro databases was conducted and randomized controlled trials (RCTs) in humans that analysed the effects of WBV in patients with ACL injury subjected to reconstruction surgery, published in English, Portuguese, Spanish, Italian, or French were included. Records were identified through database search and reference screening by two reviewers, which independently examined titles and abstracts and irrelevant studies were excluded based in eligibility criteria. Relevant full texts were analysed for eligibility, and all relevant studies were included in the systematic review.

**Results:**

Ten studies were included in the systematic review with a mean methodological quality score of 6. Results demonstrate positive effects of WBV in relevant outcomes such as knee function, electromyographic activity, balance, and muscle strength.

**Conclusions:**

WBV demonstrated a positive effect in strength, balance, electromyographic activity, and knee function.

## 1. Introduction

The knee is a complex joint that is mobile, flexible, strong, and resistant, responsible to support the body mass, that allows to be engaged in a wide range of movements and activities. Injuries in this joint and related structures greatly impair daily activities. The anterior cruciate ligament (ACL) is one of the cruciate ligaments responsible to stabilize the knee [1–3] during flexion and extension, in which the ACL and the posterior cruciate ligament act contributing to the prevention of excessive forward or backward movements of the tibia in relation to the femur, and providing rotational stability in the knee [4].

ACL rupture is a common sports-related injury that requires proper rehabilitation interventions aiming the complete recovery of the athlete [1, 5]. The annual incidence of ACL injuries is about 70 per 100,000 person-years and the costs to treat these patients arise to $7.6 billion when treated with reconstruction surgery [6, 7].

The causes of ACL sprains or ruptures are multifactorial, and this injury is the most prevalent sport injury in the knee [6, 8]. It is possible to consider that the impairment of the ACL occurs during activity/sports with sudden changes in the direction of movement, jumping and landing abnormally, rapid stopping, a stroke directly in the lateral side of the knee, or slowing down while running [1]. Symptoms of the ACL injuries include pain, tenderness along the joint line, and swelling, decreased or loss of range of motion, and difficulty to the ambulation [1]. The weakness of the knee extensor muscles has been described as one of the major concerns in the rehabilitation after ACL injury [9]. The mechanisms related to the loss of muscle strength due to ACL injuries are not well understood [10], and depending on the severity of the injury, the individuals are referred to an orthopaedic physician to verify the treatment options, including surgery, or to a physiotherapist for rehabilitation interventions [1].

Surgical repair and reconstruction tend to be the option to athletes and individuals who are younger and more active. Moreover, surgical repair/reconstruction can be also an option for those with important instability of the knee [1]. An important factor contributing to weakness after ACL injury is a failure in voluntary activation of the knee extensors independently of structural damage to the muscle or motoneurons. It is suggested that abnormal afferent discharge from the knee may modify the excitability of reflex pathways within the spinal cord. In consequence, this would lead to a reduction of the excitability of the *α*-motoneuron of the knee extensors [11]. Different interventions, either open or closed kinetic chain exercises, have been proposed to deal with muscle weakness in individuals with deficient ACL [12, 13].

A type exercise that can be used to an effective management of individuals with ACL injuries comprises the use of mechanical vibration generated in a vibrating platform (VP) that is transmitted to an individual standing over the VP. This modality is called whole-body vibration (WBV) [14–16].

The interest in the clinical application of WBV exercise is increasing, and it is believed that WBV can improve strength in the lower limb muscles [17, 18]. The authors demonstrated that the enhanced muscle contraction during WBV would be evoked via the stretch reflex pathway [12, 13]. Indeed, acute changes in motor output have been related to the increased sensitivity of muscle spindles [17, 19]. The neuromuscular response to WBV would depend on the type, frequency, peak-to-peak displacement, peak acceleration, and duration of the intervention with mechanical vibration as well as on the adopted body position on the VP [20]. The frequency of the mechanical stimulus has received increased attention [21, 22]. Cardinale and Lim [21] described a rise in neuromuscular activation of the vastus lateralis (VL) muscle when frequency increased up to 30 Hz, which was followed by a decrease in activation as WBV frequency increased. Marín et al. [23] reported that the magnitude of the WBV effect was higher with the amplitude of 4 mm in comparison to 2 mm for the VL and gastrocnemius medialis muscles. This neuromuscular activation can be of interest in individuals with ACL injuries and previous research has evidenced beneficial effects in strength, balance, and electromyographic activity (e.g., [15]); however, no systematic analysis of the existing literature about this topic has been conducted.

Considering this rationale, the aim of this systematic review is to assess the effect of WBV in the rehabilitation of patients with reconstructed anterior cruciate ligament (RACL).

## 2. Methods

The review was reported based on the Preferred Reporting Items for Systematic Reviews and Meta-Analysis (PRISMA) guidelines [24].

### 2.1. Eligibility Criteria

We considered randomized controlled trials (RCTs) in humans that analysed the effects of WBV in patients with ACL injury subjected to reconstruction surgery, if the effects of WBV could be isolated from concomitant interventions, if focusing on the effects of WBV in muscle strength, balance, postural stability, proprioception, electromyographic activity, and functionality, and if published in English, Portuguese, Spanish, Italian, or French. No publication date restrictions were defined.

### 2.2. Operational Definitions

WBV was defined as an exercise intervention consisting of the application of sinusoidal vibration to individuals using specialized vibrating platforms. These platforms deliver vibration to the whole body using two different systems, uniform movements of the platform up and down and side alternating displacements on the left and right side of a fulcrum [25].

### 2.3. Search Strategy

We conducted an electronic search in Pubmed, Scopus, Web of Science, and PEDro databases using the following search string ((“whole-body vibration” OR “whole body vibration”) AND (“anterior cruciate ligament” OR ACL)). Secondary searches were conducted on the reference lists and citation tracking of included studies to identify other possible relevant studies. The keywords used in the search were defined based on the PICO strategy, focusing on patients with RACL (Participants) receiving WBV intervention (Intervention) without restrictions regarding comparisons (Comparison), allowing comparisons to placebo, usual care or no intervention. All reported outcomes (Outcomes) were allowed if considered relevant to the studied population.

### 2.4. Study Selection and Data Extraction

All references were exported to a data management software (EndNote X9), and duplicates were removed. The review was conducted following four steps. Records were identified through database search and reference screening (Identification) and two reviewers (AS, MB-F) independently examined titles and abstracts and irrelevant studies were excluded based in eligibility criteria (Screening). Relevant full texts were analysed for eligibility (Eligibility), and all relevant studies were included in the systematic review. The disagreement was resolved by a third reviewer (DS-C).

The same researchers were responsible for data extraction from the included studies. Data regarding study information (author and year), study design and time of follow-up, subjects (sample size), demographics (age, sex, Body Mass Index), type of graft, intervention protocols, WBV intervention, outcomes, and results were extracted.

### 2.5. Methodological Quality and Risk of Bias

Two reviewers (AS, BS) used the PEDro scale [26, 27] to assess the methodological quality and the Cochrane Collaboration's tool to assess the risk of bias of the included studies [28].

## 3. Results

**Figure 1:**
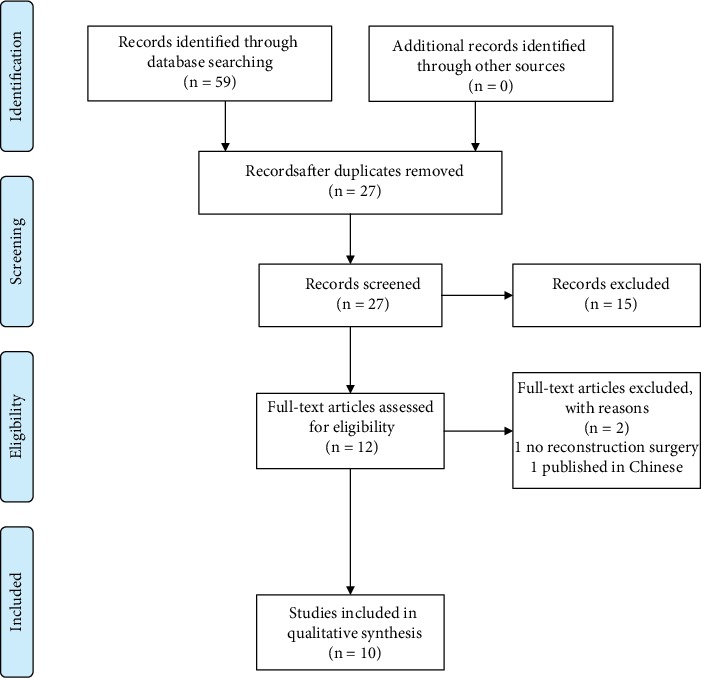
PRISMA flow diagram of the literature selection process.

A total of 59 studies were identified through a database search and, after the removal of duplicates, 27 studies were identified. During the screening process, 15 publications were excluded for not being related to the research question, and the full text of 12 studies was reviewed in detail. After careful analysis, 2 studies were excluded (1 because the subjects were not subjected to a reconstruction surgery and 1 because it was published in Chinese). Finally, 10 studies were included in the systematic review. The selection process is schematized in Figure 1.

**Figure 2:**
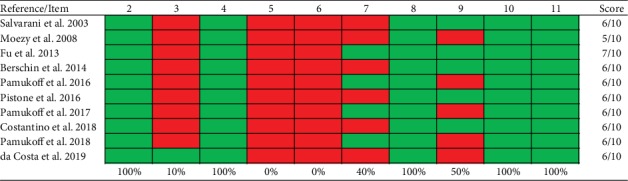
Methodological quality assessment of the included studies with PEDro scale. (2) Subjects were randomly allocated to groups (in a crossover study, subjects were randomly allocated an order in which treatments were received); (3) allocation was concealed; (4) the groups were similar at baseline regarding the most important prognostic indicators; (5) there was blinding of all subjects; (6) there was blinding of all therapists who administered the therapy; (7) there was blinding of all assessors who measured at least one key outcome; (8) measures of at least one key outcome were obtained from more than 85%; of the subjects initially allocated to groups; (9) all subjects for whom outcome measures were available received the treatment or control condition as allocated or, where this was not the case, data for at least one key outcome was analysed by “intention to treat”; (10) the results of between-group statistical comparisons are reported for at least one key outcome; (11) the study provides both point measures and measures of variability for at least one key outcome.

The included studies had a mean score of 6 when assessing the methodological quality with the PEDro scale (Figure 2), with a minimum of 5 points and a maximum of 7, evidencing moderate methodological quality.

Detailed description and results of the included studies are presented in Table 1. Seven out of ten studies were designed as randomized controlled trials [14, 15, 29–33], and three were designed as randomized crossover trials [16, 34, 35]. Four studies analysed the effects of a single session of WBV [16, 34, 35], and six studies analysed the effects of WBV programs with a minimum of two weeks (10 sessions) [33] and a maximum of ten weeks (30-40 sessions) [29]. Four studies investigated the effects of WBV and other programs [14, 30, 32, 33], and six investigated the effects of WBV alone [15, 16, 29, 31, 34, 35]. Only three studies had follow-up assessments at 1 week after the intervention [33], 3 months after the intervention [30], and one month after the intervention [32].

Most studies included male and female patients; however, Moezy et al. [31] and da Costa et al. [15] included only male subjects, Costantino, Bertuletti, and Romiti [14] have included only female participants and [32] have not stated the gender of the participants. In general, the sample size was small, ranging between 20 participants [16, 33–35] and 48 participants [30]. Studies evidenced a low attrition rate.

### 3.1. WBV Protocols

The WBV intervention protocols were heterogenous. Intervention varied in duration (as stated before), in training frequency, session duration, number of repetitions, amount of rest between repetitions, in frequency, amplitude, type of vibration (synchronous and site alternating), modality of exercises included (static or static and dynamic), and number of exercises per session. Patients were standing in all studies, with knee flexion varying from “slight” to 60° of flexion. Parameters were fixed during the protocol in some studies and varying in others, mostly based on time criteria. The timing of implementation of the WBV protocol greatly varied between studies, starting between 2 weeks [29] and 50.6 ± 21.3 months [34, 35] after surgery.

A summary of WBV protocols can be found in Table 1.

**Figure 3:**
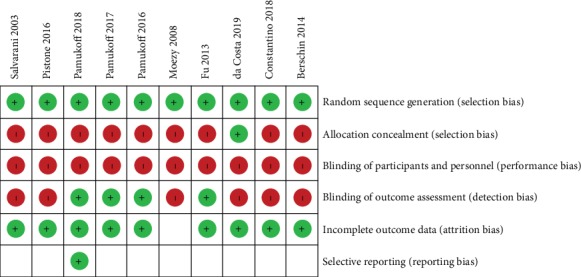
Risk of bias summary: authors assessment for each risk of bias criterion.

The risk of bias of included studies was assessed with the Cochrane risk of bias tool (Figure 3).

### 3.2. Assessed Outcomes

Several outcomes were assessed: running biomechanics (1 study: [16]), functional tests [30], Corticomotor excitability (1 study: [34]), active range of motion (1 study: [29]), motor neuron pool excitability (1 study: [34]), joint position sense (2 studiess: [30, 31]), joint laxity (2 studiess: [29, 30]), Lysholm score (2 studiess: [29, 32]), surface electromyographic signal (3 studiess: [15, 34, 35]), balance or postural stability (5 studiess: [15, 29–32]), and muscle strength (8 studiess: [14, 15, 29, 30, 32–35]).

#### 3.2.1. Running Biomechanics

A single session of WBV improved knee flexion excursion during running in the injured limb [16]. The other outcomes assessed in the study, such as loading rate, peak knee flexion angle, peak knee flexion moment, and peak vertical ground reaction force have not significantly changed. The study also suggests that the improvement was higher in patients with more impairment at baseline.

#### 3.2.2. Functional Tests

Fu et al. [30] described a significant improvement in the shuttle run test in the WBV group and in the single-legged hop test in the reconstructed limb in both groups. The WBV group also evidenced better limb symmetry during the tests. In the tests Carioca and triple hop, no significant differences were found between the groups.

#### 3.2.3. Corticomotor Excitability

The motor-evoked potential amplitude has not changed after one session of WBV but significant changes in an active motor threshold occurred [34].

#### 3.2.4. Active Range of Motion

Berschin et al. [29] reported an increment in active range of motion to full amplitude after the WBV protocol but with no significant difference between the groups.

#### 3.2.5. Motor Neuron Pool Excitability

Regarding *H*-reflex and maximal muscle response (*M*-wave), in the study of Pamukoff et al. [34], no significant differences were found after the intervention.

#### 3.2.6. Joint Position Sense

Moezy et al. [31] reported significant improvements in proprioceptive acuity after WBV intervention in both testing amplitudes 30° and 60° of knee flexion and both limbs (injured and uninjured) except in uninjured knee at 30°, but not in the control group. However, Fu et al. [30] reported no significant differences in proprioceptive acuity when repositioning to 30° and 60° of knee flexion after intervention and between groups in both lower limbs.

#### 3.2.7. Joint Laxity

Both ([29, 30] reported no significant side-to-side differences in joint laxity in WBV and control groups before and after the intervention.

#### 3.2.8. Lysholm Score

Berschin et al. [29] reported that knee function improved in both WBV and control group, with no significant differences between the groups but [32] reported significantly higher functional gains in the treatment group both at post-intervention and follow-up.

#### 3.2.9. Surface Electromyographic Signal

Pamukoff et al. [34] reported significant changes in quadriceps electromyographic amplitude but not in the hamstrings after WBV, and electromechanical delay has not changed after intervention in the study of Pamukoff et al. [35]. However, in the study of da Costa et al. [15], the electromyographic amplitude of the *vastus lateralis* and *vastus medialis* has not changed after intervention.

#### 3.2.10. Balance or Postural Stability

Moezy et al. [31] observed a significant improvement in overall stability, anteroposterior, and mediolateral indexes in both opened and closed eyes conditions after WBV, which was statistically greater than in the control group. Fu et al. [30] have also reported significantly higher improvements in overall, anteroposterior and mediolateral stability indexes in the WBV group both after intervention and at follow-up (3 months). Berschin et al. [29] have also reported significant improvements in stability index after WBV, but not in the control group. Significant changes between groups were observed in the 8^th^ and 11^th^ weeks. Another study reported significant improvements in stability in balance tests with eyes opened, with better results in the WBV group, but not with eyes closed. However, in eyes closed tests at follow-up, the WBV group performed better [32]. The study of da Costa et al. [15] was the only not reporting significant improvements after intervention with WBV.

#### 3.2.11. Muscle Strength

In studies analysing the effects of a single WBV session, Pamukoff et al. [34] reported significant improvements in central activation ratio and knee extensor peak torque but no significant changes in the rate of torque development. However, the same authors reported that WBV induced a significant increase in early (0-100 ms) rate of torque development but not in late (100-200 ms) rate of torque development [35]. Finally, da Costa et al. [15] found no significant differences in knee extensor peak torque after a single WBV session.

In studies analysing the effects of WBV programs with several sessions, Salvarani et al. [33] reported an increase in knee extensor strength in both WBV and control groups, but an increase in the mid-second of contraction only occurred after WBV. Fu et al. [30] observed an increase in knee extensor strength in the reconstructed limb in all analysed velocities (60°/s, 180°/s, and 300°/s) at the 3-month follow-up assessment, when compared to the control group, and an increase in flexor strength in the reconstructed limb at 60°/s, when compared to the control group. Three months after the rehabilitation program, only the WBV group had higher peak torque at 300°/s. Berschin et al. [29] verified an increase in extensor and flexor strength in both control and WBV groups. In the 11^th^ week, isometric extensor strength was significantly higher in the control (conventional strengthening program) group. In another study, limb symmetry index in knee extension maximal voluntary contraction (MVC) increased significantly in both WBV and control group, and limb symmetry index in knee flexor MVC increased significantly in the WBV group at postintervention and follow-up [32]. Finally, Costantino, Bertuletti, and Romiti [14] reported significant improvements in peak torque and maximum power in knee extensors and flexors, with significantly higher improvements in the WBV group.

## 4. Discussion

The main goal of this systematic review was to assess the effect of WBV in the rehabilitation of patients with RACL. After analysing the included studies and considering their limitations, the results suggest that WBV may be a valid intervention in this population.

### 4.1. Methodological Quality of the Studies

The methodological quality of the included studies was moderate. Regarding concealed allocation, only one study stated the use of numbered, sealed, and opaque envelopes that were only opened at the moment of the intervention [15]. None has blinded participants and therapy administrators and only four referred blinded assessors in outcome measurement [16, 30, 34, 35]. The impact of these issues is well known and discussed [36].

### 4.2. Effects on Running Biomechanics, Functional Tests, Active Range of Motion, Corticomotor, and Motor Neuron Pool Excitability

Positive findings in these outcomes were reported. However, the limited number of studies addressing each of the outcomes limits the ability to establish its relevance to clinical practice. More studies, with high methodological quality, are needed to address these effects in the future and allow to establish solid recommendations.

### 4.3. Effects on Joint Position Sense

Conflicting evidence exists in this outcome. Moezy et al. [31] reported a significant increase in proprioceptive acuity after the WBV intervention; however, Fu et al. [30] found no significant changes after the WBV protocol. Both studies assessed joint position sense with an isokinetic dynamometer, but the assessment modality was different. Moezy et al. [31] used active repositioning during the assessment, and Fu et al. used passive repositioning in the assessment. This difference alone may explain the divergence in outcomes. Active repositioning is known to increase the activation of muscle receptors when compared to passive repositioning [37]. WBV may increase muscle activity in knee extensors [34] which may be related to a higher activity of muscular mechanoreceptors, contributing to a decrease in absolute errors while assessing joint position sense actively. The WBV protocol was similar in both studies, but the timing of the implementation was different. Fu et al. [30] started 1 month after surgery and Moezy et al. [31] started 12 weeks after surgery, which also may have contributed to the observed differences. However, more studies are needed to understand the impact of the modality of limb repositioning and the timing of protocol implementation in proprioceptive acuity outcomes.

### 4.4. Effects on Joint Laxity

Two studies [29, 30], using different kinds of vibration—vertical synchronous and side alternating, respectively—failed to evidence significant changes in side-to-side differences in joint laxity, assessed with the KT 1000 arthrometer, in both WBV and conventional or standard rehabilitation groups. However, no study has assessed the effects of WBV in dynamic joint stability, which is a functionally more interesting parameter and should be addressed in future research.

### 4.5. Effects of Knee Function

Two studies [29, 32] have found that WBV increases knee function. However, when compared to the respective control groups (standard strengthening program and traditional rehabilitation program), only the study of Pistone et al. [32] reported significantly higher functional improvement in the WBV group. Several aspects can justify this difference, the nature of the control group, the timing of implementation, and the WBV parameters. Berschin et al. [29] compared the effects of the WBV to a group performing a standard strengthening program and this focus on strengthening may have contributed to the lack of differences between the groups as strength is a key parameter to increase functional status after ACL injury [38]. On the other hand, the timing of implementation of the WBV program may have been an important factor. Berschin et al. [29] started the WBV program 2 weeks after surgery, and Pistone et al. [32] started the program 1 month after surgery. Considering the natural differences in functional status between 2 weeks and 1 month after surgery this may have played an important role in the differences in outcomes between the studies. However, further research should analyse the impact of the timing of implementation of the WBV program in functional status. Finally, the differences in the WBV program parameters should be discussed. Pistone et al. [32] have not used a fixed WBV frequency, but rather the optimal vibration frequency was previously determined, which may explain why results in the WBV group were significantly better. The optimal vibration frequency has been defined as the vibration frequency at which maximal muscle activation arises, and according to Giombini et al. [18], it is advisable to prescribe individualized vibration parameters to maximize the improvement in outcome measures.

### 4.6. Effects on Surface Electromyographic Signal

More research is needed to understand the effect of WBV on parameters related to the electromyographic activity of knee extensors and flexors. No study has assessed the long-term effects of WBV on electromyographic activity in patients with RACL, and only three studies have analysed the acute effects of WBV in parameters in this domain, but the parameters and/or methodologies were distinct. Pamukoff et al. [34] reported a significant increase in the electromyographic amplitude of knee extensors but not in knee flexors, and da Costa et al. [15] reported no significant changes in knee extensor electromyographic amplitude. Previous research suggested that higher vibration frequencies and amplitudes elicited the highest changes in electromyographic signal [39, 40]. However, the studies of da Costa et al. [15], using a frequency of 50 Hz, and Pamukoff et al. [34], using a frequency of 30 Hz, suggest the opposite. It should be noted that the study populations are different, and the studies included in this review addressed a clinical population, which can suggest that healthy subjects and subjects with a condition may benefit differently from WBV. However, regarding the differences in outcomes of Pamukoff et al. [34] and da Costa et al. [15], it should be stressed that the timing of implementation of the WBV program is different, 50.6 ± 21.3 months and 16.8 ± 1.55 weeks after surgery, respectively, suggesting that early implementation may lead to better results in these parameters. WBV does not seem to decrease the time between the onset of the surface electromyographic signal and the onset of torque in knee extensors and flexors; however, as stated before, only one short-term study [35] has addressed this question, and more research is needed to increase the body of knowledge on the topic.

### 4.7. Effects on Balance or Postural Stability

Previous research has established that WBV training could enhance muscle spindle sensibility and excitability, which could lead to reduced reaction time of postural muscles and motor unit recruitment thresholds [17, 41] and that lower WBV frequencies could be more beneficial when training balance [42].

There is a clear positive effect of WBV training on balance in patients with RACL. Out of five studies analysing this effect [15, 29–32] only one, analysing the acute effects of WBV training, failed to provide positive effects on balance [15]. This suggests that a single session of WBV may be insufficient to elicit positive adaptations in the neuromuscular system, and this should be noticed by clinicians. Another interesting aspect is that positive effects occurred when lower [29], higher [30, 31] or custom [32] vibration frequencies were employed, and the same is true for WBV amplitude.

### 4.8. Effects on Muscle Strength

Two types of research articles analysed the effects of WBV on muscle strength, those assessing the effects of a single training session and those assessing the effects of several WBV sessions.

Concerning improvements knee extensor peak torque and rate of torque development. Pamukoff et al. [34] described significant improvements in knee extensor peak torque, contrary to the findings of da Costa et al. [15] that found no significant differences in peak torque after a single session of WBV. The differences in the timing of protocol implementation and protocol parameters, especially vibration frequency, may explain the discrepancy as Tseng et al. [42] states that immediate neuromuscular function is impaired when vibration frequency exceeds 40 Hz. Only the rate of torque development in the first 100 ms maximal isometric knee extension contraction seems to improve after a single session of WBV [34, 35]. Often, dynamic tasks require force production before 300 milliseconds, but early torque production, during the first 100 milliseconds, may be a more reliable parameter for functional tasks in which the knee extensors must produce submaximal levels of force rapidly, such as immediately before ground contact during gait to attenuate the vertical ground reaction force [43].

Regarding muscle strength improvements after WBV programs with several training sessions, adding WBV to standard/conventional treatment programs [14, 30, 32, 33] provides important benefits providing better resistance to fatigue, increasing the mid-second of contraction, increasing performance in knee extensors and flexors, increasing limb symmetry indexes in knee extensors and flexors and higher improvements in peak torque and maximum power in knee extensors and flexors. Only the study of Berschin et al. [29] failed to demonstrate better results in the WBV group. This was also the only study where WBV was used alone against a control group performing a standard strengthening program, which achieved significantly higher isometric knee extensor strength at the 11^th^ week. These findings suggest that WBV should be used as a complement to rehabilitation programs to provide significantly better results.

However, considering the heterogeneity in WBV protocols, more research is needed to identify the optimal protocol to be implemented in patients with RACL to improve neuromuscular function.

### 4.9. Adverse Effects

Only one study [29] reported minor complications such as pain or swelling during or after WBV exercise in 12/20 (60%) participants up to the sixth week but in the control group the same complications occurred in 14/20 (70%).

### 4.10. Limitations

The findings of this systematic review must be interpreted with caution. Although four well-known databases were used, including more sources of data could have improved the amount of literature included in the review. The same goes for the search terms that, although inclusive, could have provided different results if a broader search strategy was used, and therefore not all relevant studies might have been identified. Moreover, within the included studies, limitations are present in terms of study design, heterogeneity of WBV protocols, heterogeneity of control groups, and cohorts. This heterogeneity makes the comparison between studies and interpretation of WBV effects very difficult. Regarding the included studies cohorts, the included trials had small sample sizes and heterogenous samples. Demographic data was not always described.

## 5. Conclusion

WBV interventions in patients with RACL evidenced high patient compliance. This training method demonstrated that it can have a positive impact in strength, balance, electromyographic activity, and knee function. Therefore, implementing WBV interventions in this population seems possible and effective in improving parameters that are relevant to patients recovering from RACL.

### 5.1. Future Research

High-quality randomized clinical trials are needed, with proper allocation concealment and blinding, and trial registration to ensure that selective reporting is not an issue. Future studies should investigate the effects of WBV, with adequate follow-up after intervention, on relevant functional parameters, and should compare the effects of different types of WBV (synchronous and side alternating) and different vibration frequencies and amplitudes, aiming to determine the best protocol for these patients.

## Figures and Tables

**Table 1:** Summary table of the included studies in the review with main findings.

Study	Study design	Demographics	Graft	Intervention protocol	WBV intervention	Follow-up after intervention	Results
Salvarani [33]	RCT	20 subjects (17 males/3 females) TG: 10 subjects (29.7 ± 7.8 years; 174.1 ± 7.7 cm; 72.0 ± 7.6 kg) CG: 10 subjects (26.8 ± 5.2 years; 175.2 ± 8.3 cm; 73.2 ± 7.9 kg)	Patellar tendon	1 month after surgery: TG: standard treatment + WBV 10 sessions 1 daily session/2 weeks CG: standard + isometrics in the same position of TG 10 sessions 1 daily session/2 weeks	Synchronous vibration Freq: 30 Hz Duration: 1 min/repetition, 5 repetitions Rest: 1 min rest/set Isometric contraction with position: Knee flexion of 25°	1 week	TG: significant increase in extensor strength after intervention. Significant increase from baseline to follow-up but not between postintervention and follow-up CG: significant increase in extensor strength after intervention. Significant increase from baseline to follow-up but not between postintervention and follow-up.

Moezy [31]	RCT	20 male subjects (23 initially, 3 dropped out during intervention, 2 in TG and 1 in CG) TG: 12 subjects (24.51 ± 3.38 years; 1.74 ± 0.05 m; 74.30 ± 10.61 kg; 24.51 ± 3.38 kg/m2) CG: 11 subjects (22.70 ± 3.77 years; 1.78 ± 0.07 m; 78.00 ± 10.12 kg; 24.62 ± 2.78 kg/m2)	Patellar tendon	12 weeks after surgery: TG: WBV 12 sessions 3x/week for 1 month CG: conventional strength, flexibility, and proprioceptive training program 12 sessions 3x/week for 1 month	Synchronous vibration Parameters changed during intervention Freq: 30-50 Hz Duration: 30-60 secs/set Rest: 30-60secs Amplitude: 2.5–5 mm Modality: static-static and dynamic Session duration: 4-16 min Position: several exercises, different knee position	None	TG: significant improvement in overall stability, anteroposterior and mediolateral indexes in opened and closed eye tests. Significant improvements in absolute angular errors in both knees in both testing amplitudes (except in healthy knee at 30°) CG: significant improvements in overall stability in opened and closed eyes and significant improvement in mediolateral index in closed eye test. No significant differences in proprioceptive acuity. The improvement in stability and proprioceptive acuity scores was significantly higher in the TG, except in absolute angular error in healthy knees at 30°

Fu [30]	RCT	48 subjects (32 males/16 females, 9 dropped out, 5 in TG and 4 in CG but were included in the analysis) TG: 24 subjects (18 males, 23.3 ± 5.2 years; 66.5 ± 12.8 *kg*; 1.71 ± 0.08 m; 22.75 ± 3.44 kg/m2) CG: 24 subjects (14 males, 25.2 ± 7.3 years; 66.7 ± 10.5 kg; 1.70 ± 0.07 m; 23.11 ± 2.84 kg/m2)	Hamstrings	1 month after surgery: TG: WBV+conventional training 16 sessions 2x/week for 2 months CG: conventional training	Synchronous vibration Parameters changed during intervention Freq: 35-50 Hz Duration of sets: 30-45 secs Rest: 15-30 secs Amplitude: 4 mm Modality: static-static and dynamic Session duration: 4-16 min Position: several exercises, different knee position	3 months	No significant differences regarding joint position sense throughout 6 months between groups, in both limbs. With eyes closed, the TG had significantly better overall anteroposterior and mediolateral stability indexes than the CG. The TG evidenced significant improvements in overall anteroposterior and mediolateral stability indexes 3 months after surgery, but the anteroposterior index significantly decreased in the CG. Reconstructed limb's knee extensors in the TG evidenced significantly higher peak torques than the CG in all velocities (60, 180, and 300°/s) at 6 months after surgery. Reconstructed limb's knee flexors in the TG evidenced significantly higher peak torques than the CG at 60 and 300°/s. 3 months after training only the TG evidenced significant improvement in knee extensors and flexors at 300°/s. The TG also evidenced better limb symmetry throughout the rehabilitation process. The TG performed significantly better in the shuttle run test. In the single-legged hop test, the reconstructed limb in both groups performed significantly better. Subjects in the TG had better limb symmetry throughout the rehabilitation process. No significant differences between both groups regarding triple hop and carioca tests. No significant differences regarding joint laxity between the two groups and both groups achieved full range of motion.

Berschin [29]	RCT	40 subjects (29 males/11 females) TG: 20 subjects (14 males; 27 ± 4.2 years; 23.2 ± 3.4 kg/m2) CG: 20 subjects (15 males; 28 ± 6.8 years; 24.3 ± 2.8 kg/m2)	Patellar tendon	2 weeks after surgery: TG: WBV exercise protocol average 40 ± 2.3 min/session Unclear number of sessions 3-4x/week for 10 weeks CG: standard strengthening protocol average 85 ± 4.4 min/session Unclear number of sessions 3-4x/week for 10 weeks	Side alternating vibration Parameters changed during intervention (3 phases) Freq: 10-30 Hz Duration of sets: 1-2 min Rest: not clearly stated Amplitude: 5-9 mm Modality: static-dynamic Position: slight knee flexion in static, varying in dynamic exercise	None	Range of motion increased in both groups to full motion with no significant differences between groups. No significant changes in joint laxity in both groups after intervention. Extensor and flexor strength improved significantly in both groups with similar results in isometric and isokinetic testing, except for isometric extensor testing at 11 weeks which was significantly higher in the CG. The TG evidenced a significant increase in stability index, but not the CG. Significant differences in this outcome between groups in the 8^th^ and 11^th^ weeks. Knee function (Lysholm score) improved significantly in both groups without significant differences between groups.

Pamukoff [35]	Randomized crossover trial	20 subjects (6 males, 21.1 ± 1.2 years; 168.4 ± 9.5 cm; 68.3 ± 14.9 kg)	16 patellar tendon 3 hamstrings 1 allograft	50.6 ± 21.3 months after surgery TG: WBV, 1 session TG2: local muscle vibration, 1 session CG: no intervention	Synchronous vibration Freq: 30 Hz Duration: 1 min/repetition 6 repetitions Rest: 2 min between repetitions Amplitude: not stated Acceleration: 2 g Modality: static Position: knee flexion 60°	None	No significant changes were observed in the rate of torque development, motor-evoked potential amplitude, *H*-reflex amplitude, hamstrings electromyographic amplitude, and quadriceps *M*-wave amplitude. Significant improvements in active motor threshold (corticomotor excitability), central activation ratio, quadriceps peak torque, and quadriceps electromyographic amplitude. Improvements after WBV were not significantly different from those observed in local muscle vibration.

Pistone [32]	RCT	34 subjects (gender not specified) TG: 17 subjects (27 ± 7 years; 1.76 ± 0.08 m; 73.3 ± 11.9 kg) CG: 17 subjects (29 ± 7 years; 1.75 ± 0.08 m; 73.0 ± 10.7 kg)	Semitendinous	1 month after surgery: TG: traditional rehabilitation program and WBV 5 days/week TRP+12 sessions, 3 sessions/week for 4 weeks, of WBV CG: traditional rehabilitation program 5 days/week TRP	Synchronous vibration Freq: optimal vibration frequency—frequency (Hz) with maximal muscle activation Duration: 1 min/repetition; number of repetitions increased during protocol from 3 to 10 Rest: 1 min between repetitions Amplitude: 2 mm Modality: static Position: knee flexion of 60°	1 month	Limb symmetry index in knee extension MVC increased significantly between baseline and postintervention and between baseline and follow-up in both groups equally. Limb symmetry in knee flexor MVC increased significantly between baseline and follow-up in the CG and between baseline and postintervention, between baseline and follow-up, and between postintervention and follow-up in the TG. LSI of knee flexion in the TG was significantly higher than CG at postintervention and follow-up. During balance trials with eyes open, significant changes were observed over time in both groups, but at follow-up, the TG performed significantly better. During balance trials with eyes closed, there were no significant changes over time, but at follow-up, the TG performed significantly better. Improvements in the Lysholm score were greater in TG than in the CG at postintervention and follow-up.

Pamukoff [35]	Randomized crossover trial	20 subjects (6 males, 21.1 [20.6-21.6] years; 168.4 [164.2-172.6] cm; 68.3 [61.8-74.8] kg) (mean [95% CI])	16 patellar tendon 3 hamstrings 1 allograft	50.6 (95% CI: 41.3-59.9) months after surgery TG: WBV, 1 session TG2: local muscle vibration, 1 session CG: no intervention	Synchronous vibration Freq: 30 Hz Duration: 1 min/repetition 6 repetitions Rest: 2 min between repetitions Amplitude: not stated Acceleration: 2 g Modality: static Position: knee flexion 60°	None	WBV, but not the other conditions, significantly increased the early rate of torque development (0-100 ms) during a maximal isometric knee extension. Late rate of torque development and electromechanical delay has not changed significantly. No differences between conditions were observed after intervention.

Costantino [14]	RCT	38 female subjects (39 initially, 1 dropped during intervention in the TG) TG: 19 subjects (25.47 ± 2.01 years; 166.16 ± 5.18 cm; 56.00 ± 3.92 kg; 20.29 ± 1.28 kg/m2) CG: 19 subjects (25.42 ± 2.39 years; 166.11 ± 5.34 cm; 55.32 ± 5.18 kg; 20.06 ± 1.80 kg/m2)	Patellar tendon	13 weeks after surgery: TG: 2 static exercises with WBV in addition to the rehabilitation protocol 3 sessions/week for 8 weeks. CG: 2 static exercises without WBV in addition to the rehabilitation protocol 3 sessions/week for 8 weeks.	Synchronous vibration Freq: 26 Hz Duration: 1 min/repetition, 6 repetitions/exercise Rest: 1 min between repetitions, 2 min between sets Amplitude: 4 mm Modality: static Position: knee flexion of 25°	None	All strength parameters (peak torque and maximum power) in knee extensors and knee flexors improved significantly in both groups. TG had a significantly higher improvement than the CG.

Pamukoff [16]	Randomized crossover trial	20 subjects (15 females, 22.3 ± 3.3 years; 173.0 ± 9.1 cm; 71.8 ± 15.3 kg)	10 patellar tendon 7 hamstrings 3 allograft	44.9 ± 22.8 months after surgery TG: WBV, 1 session CG: no intervention	Synchronous vibration Freq: 30 Hz Duration: 1 min/repetition 6 repetitions Rest: 2 min between repetitions Amplitude: not stated Acceleration: 2 g Modality: static Position: knee flexion 60°	None	WBV vibration significantly increased knee flexion excursion in the injured limb but not loading rate, peak knee flexion angle, peak knee flexion moment, and peak vertical ground reaction force. Subjects with more baseline impairment had larger changes in knee flexion excursion.

da Costa [15]	RCT	44 male subjects (48 initially, 4 dropped out during evaluation due to fatigue and/or discomfort, before intervention) TG: 22 subjects (28.0 ± 5.52 years; 1.75 ± 0.79 m; 27.1 ± 4.49 kg/m2) CG: 22 subjects (26.8 ± 6.83 years; 1.74 ± 0.63 m; 26.5 ± 2.96 kg/m2)	Gracilis-semitendinosus	Postoperative time: TG: 16.8 ± 1.55 weeks CG: 17.0 ± 1.26 weeks TG: WBV squat 1 session CG: squat without vibration	Synchronous vibration Freq: 50 Hz Duration: 10 sets of 30 secs Rest: 30 secs between sets Amplitude: 4 mm Modality: static Position: knee flexion of 40°	None	No significant improvements in any of the outcome variables (peak torque, total work, electromyographic activity, and oscillation of the centre of pressure) and no significant differences between groups were observed.

RCT: randomized controlled trial; COP: centre of pressure; Freq: frequency; CG: control group; LSI: limb symmetry index; MVC: maximal voluntary contraction; TG: training group; ROM: range of motion; TRP: traditional rehabilitation program; WBV: whole-body vibration.
